# Triptolide Induces Leydig Cell Apoptosis by Disrupting Mitochondrial Dynamics in Rats

**DOI:** 10.3389/fphar.2021.616803

**Published:** 2021-03-09

**Authors:** Linyan Lv, Yajie Chang, Yanqing Li, Haicheng Chen, Jiahui Yao, Yun Xie, Xiaoyan Liang, Xing Yang, Min Zhang, Guihua Liu

**Affiliations:** ^1^ Reproductive Medicine Research Center, The Sixth Affiliated Hospital of Sun Yat-sen University, Guangzhou, China; ^2^ Department of Andrology, The First Affiliated Hospital of Sun Yat-sen University, Guangzhou, China

**Keywords:** triptolide, leydig cells, DRP1, mitochondrial dynamic, apoptosis

## Abstract

Triptolide is widely used in the clinical treatment of various diseases. Side effects, including reproductive toxicity to male patients, limit its application. However, no detailed mechanisms or potential intervention targets have been reported. In this study, we show that triptolide activated the mitochondrial apoptosis pathway in rat testicular Leydig cells and induced apoptosis both *in vivo* and *in vitro*, which may cause hypoleydigism and impair spermatogenesis. Mechanistically, triptolide-induced dynamin-related protein 1 (Drp1) overexpression, which interfered with mitochondrial dynamic stability to activate the mitochondrial apoptosis pathway. Mdivi-1, a selective Drp1 inhibitor, partially reversed the mitochondrial dynamic disturbance and rat testicular Leydig cell apoptosis induced by triptolide. Inhibiting Drp1 over-activation may be a new strategy for mitigating the reproductive toxicity of triptolide.

## Introduction

Triptolide (TP), an active compound from a Chinese herb, is now widely used in the clinic to treat multiple diseases, including autoimmune diseases, cancers, and diabetic nephropathy. With the incidences of the diseases above increasing among males, the side effects of triptolide, especially its reproductive toxicity, limit its application (Du et al., 2018). Xiong et al. reported that triptolide decreased testicular weight, damaged testis and sperm morphology, and reduced sperm motility and density (Xiong et al., 2019). Wang et al. reported that triptolide induced oxidative stress and apoptosis in TM4 cells (Wang et al., 2018). These studies provide some clues on the reproductive toxicity of triptolide. However, more detailed mechanisms and therapeutic targets are still needed.

Testicular Leydig cells nourish the spermatogonium and provide testosterone, which is important for spermatogenesis. Mitochondria are vital for Leydig cell survival and steroidogenesis (Park et al., 2019). Triptolide causes mitochondrial membrane depolarization and activates the mitochondrial apoptosis pathway in murine pituitary corticotroph tumor cells (Li et al., 2017b). Whether triptolide damages Leydig cell mitochondria and decreases their function in spermatogenesis is still not clear.

Mitochondrial fission and fusion maintain a dynamic balance to preserve normal mitochondrial function and adapt to environmental changes (Scott and Youle, 2010). Disruption of this balance leads to mitochondrial dysfunction, which occurs in various diseases (Mouli et al., 2009; Haun et al., 2013; Khacho et al., 2014). Dynamin-related protein 1 (Drp1) is a major factor that promotes mitochondrial fission to regulate mitochondrial shape and function (Smirnova et al., 2001; Simula et al., 2019). Aberrant Drp1 expression disrupts the balance of mitochondrial fission and fusion, causing various diseases, including Parkinson’s disease, pancreatic tumors, and Huntington’s disease (Wang et al., 2011; Haun et al., 2013; Nagdas et al., 2019; Qi et al., 2019). Here, we investigated triptolide-induced aberrant Drp1 expression in Leydig cells, which disrupted the dynamic balance of mitochondrial fission and fusion, damaged mitochondrial function and finally induced steroidogenesis impairment *in vivo* and *in vitro*.

## Materials and Methods

### Chemical Agents

Triptolide (TP; cat. no. HY-32735) and mitochondrial division inhibitor 1 (Mdivi-1; cat. no. HY-15886) were purchased from MedChemExpress. Briefly, 1 mg TP was dissolved in 2.7747 ml dimethyl sulfoxide (DMSO) to prepare stock solutions (1 mM). *In vitro*, the TP stock solution was diluted with culture medium to make the working solution. *In vivo*, TP was intragastrically administered at a dose of 400 μg/kg for 0–8 weeks in different groups of rats. Ten milligrams of Mdivi-1 powder was dissolved in 2.8311 ml DMSO to prepare stock solutions (10 mM), and 1 µl stock solution was added to 1 ml culture medium to make the working solution (10 µM).

### Animal Studies

Adult male Sprague–Dawley rats (12 weeks old) were purchased from the Animal Center of Sun Yat-Sen University (Guangzhou, China). Animal studies were performed in accordance with the recommendations from the Guide for the Care and Use of Laboratory Animals of the National Institutes of Health and the Animal Welfare Act guidelines. The protocol of the present study was approved by the Ethical Committee of Sun Yat-Sen University (Guangzhou, China). Seventy-two rats were randomly divided into nine groups (eight per group) that were treated with triptolide for different periods (0–8 w). After 8 weeks, the blood and testes of rats from the different groups were collected.

### Histological Analysis

Isolated rat testes were fixed with 4% paraformaldehyde for paraffin embedding and sectioned at 3 μm thickness for tissue slices. Other rat testis tissues were stored in liquid nitrogen for subsequent experiments. The tissue slices were stained with hematoxylin and eosin (H&E) using commercial kits (Servicebio, Wuhan, China) according to the manufacturer’s instructions. The slices were examined by electron light microscopy (Olympus, Japan).

### TUNEL Assay

Apoptosis rates in rat testis tissue were tested using a terminal deoxynucleotidyl transferase–mediated dUTP nick-end labeling (TUNEL) staining kit (Thermo Fisher Scientific, MA, United States) according to the manufacturer’s instructions. After staining, the images were analyzed by confocal microscopy (Leica TCS SP8; Leica Microsystems, Inc.).

### Cell Culture

The testis Leydig cell line TM3 was purchased from the Cell Bank of the Chinese Academy of Sciences (cat. no. GNM24). Cells were cultured in RPMI-1640 medium (cat. no. 12633012; Gibco; Thermo Fisher Scientific, Inc.) containing 5% fetal bovine serum (FBS; cat. no. 10099141C Gibco; Thermo Fisher Scientific, Inc.) and 2.5% horse serum (cat. no. 26050088 Gibco; Thermo Fisher Scientific, Inc.) in a humidified incubator with 5% CO_2_. After growing to 60–70% confluency, the cells were transferred to 96- or 6-well plates for different assays.

### Cell Apoptosis Detection

7-Aminoactinomycin D (7-AAD) and Annexin V-allophycocyanin (APC) flow cytometry assays (cat. no. 70-AP105-100; MultiSciences) were used to detect cell apoptosis according to the manufacturer’s instructions. Briefly, cells were harvested after different treatments. After washing with cold PBS buffer three times, the cell pellets were resuspended in binding buffer and stained with APC-conjugated Annexin V and 7-AAD for 10 min. Then, the cells were analyzed by flow cytometry (FACS Canto II; Becton, Dickinson and Company).

### Cell Viability Detection

A total of 2,000 cells per well were seeded into 96-well plates. After culturing for 12 h, different treatments were added according to the corresponding experimental conditions. Then, 10 μl of Cell Counting Kit-8 reagent (CCK-8; cat. no. CK04; Dojindo Molecular Technologies, Inc.) was added to each well and incubated for an additional 1.5 h. The optical density values were measured at a 450-nm wavelength on a microplate reader (Multiskan^TM^ FC; Thermo Fisher Scientific, Inc.).

### ROS Assay

CellROX Oxidative Stress Reagents (cat. no. C10443; Thermo Fisher Scientific, Inc.) were used to detect cell ROS according to the manufacturer’s instructions. The cells were incubated with serum-free medium containing 5 µM CellROX Reagent for 30 min at 37°C after different treatments and then washed with PBS three times. The cells were observed under a laser scanning confocal microscope (Leica TCS SP8; Leica Microsystems, Inc.).

### Mitochondrial Membrane Potential (MMP) Assay

Tetramethylrhodamine (TMRM; cat. no. I34361; Thermo Fisher Scientific, Inc.) was used to detect MMP according to the manufacturer’s instructions. Briefly, cells in different groups were stained with 50 nmol/l TMRM in serum-free medium at 37°C for 30 min, washed with PBS three times, and then observed under a laser scanning confocal microscope (Leica TCS SP8; Leica Microsystems, Inc.) or analyzed by flow cytometry (FACS Canto II; Becton, Dickinson and Company).

### Western Blot Analysis

TM3 cells (10^6^ cells) or 20 mg testicular tissue was added to 100 µl radioimmunoprecipitation assay lysis buffer (cat. no. P0013B; Beyotime Institute of Biotechnology) with phenylmethanesulfonyl fluoride (cat. no. ST506; Beyotime Institute of Biotechnology). The concentrations of cell lysates were quantified by a Pierce BCA Protein Assay Kit (cat. no. 23227; Thermo Fisher Scientific, Inc.). Antibodies against Drp1, Mfn1, Fis-1, OPA1, and Bcl-2 were purchased from Abcam (cat. nos. ab184247, ab106274, ab157457, and ab59348). Antibodies against Bax, cleaved caspase 3, and caspase 3 were purchased from Cell Signaling Technology (cat. nos. 5023, 9661, and 9662, respectively). Antibodies against GAPDH were purchased from ProteinTech Group, Inc. (cat. no. 10494-1-AP). HRP-conjugated secondary antibody was obtained from CWBIO (cat. no. CW0103).

### Blood Sample Collection and ELISA

Blood samples were collected from the abdominal aorta after triptolide or placebo administration. After incubation at room temperature for 1 h, the blood samples were centrifuged at 1,500 rpm for 20 min, and the upper serum was collected. Serum testosterone levels of rats were detected by using a Rat Testosterone ELISA Kit (cat. no. CSB-E05100r; Cusabio) according to the manufacturer’s instructions.

### Immunohistochemistry

The protein level of Drp1 in testis tissue was detected as described before. Briefly, testis tissue slices from different rats were incubated with anti-Drp1 (1:150, ab184247, Abcam) antibody overnight at 4°C followed by incubation with a corresponding horseradish peroxidase–conjugated secondary antibody and hematoxylin staining. Randomly selected sections of the tissue slices were examined using light microscopy (Olympus, Japan).

### Statistical Analysis

Data represent the mean ± SD from three independent experiments and were analyzed using SPSS 25.0 (IBM Corp.). One-way analysis of variance followed by Tukey’s post hoc test was used to measure differences between groups. A difference with a *p* value less than 0.05 was considered statistically significant.

## Result

**FIGURE 1:**
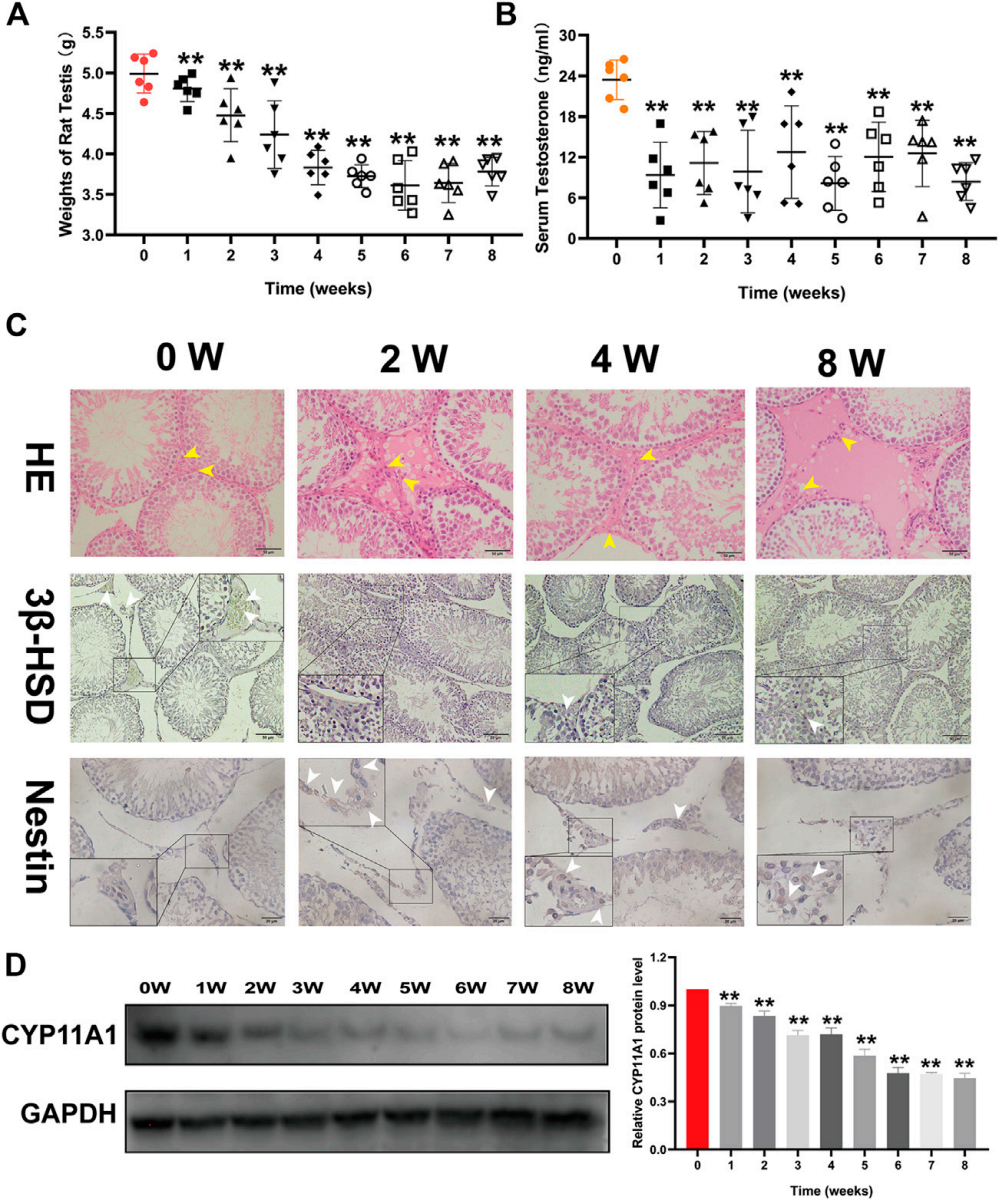
Triptolide damages the interstitial tissue of rat testes and decreases serum testosterone levels in rats. **(A)** Weights of rat testes in each group (*n* = 6/group) were measured. **(B)** Serum testosterone levels of rats in each group (*n* = 6/group) were detected using an ELISA kit. **(C)** First line: Representative images of HE-stained rat testes. Second line: Representative images of 3β-HSD immunostaining in rat testes. Third line: Representative images of nestin immunostaining in rat testes. **(D)** Western blot analysis of CYP11A1 protein levels in rat testes. Data represent the mean ± SD (*n* = 6/group) for panels **(A,B)**. Data represent the mean ± SD from three independent experiments for panels **(C,D)**. Group comparisons were performed by one-way analysis of variance followed by Tukey’s post hoc test. **p < 0.01 vs. the 0-week group. *p < 0.05 vs. the 0-week group. White arrows indicate positively stained cells in the IHC assay. Yellow arrows indicate cells in the interstitial tissue of rat testes.

1. Triptolide damages rat testes and decreases testosterone synthesis–associated enzyme levels.
To identify the effect of triptolide on rat testes, we measured testicular weights, diameters, and serum testosterone levels of rats in different groups. We found that the weights and testosterone levels were both reduced after triptolide treatment (Figure 1A,B). However, no significant difference was observed in diameter (SupplementaryFigure S1). Then, we conducted HE staining of rat testes. The results showed obvious structural disorder, including decreased cell numbers and vacuolation in the interstitial tissue of rat testes after 2 weeks of triptolide treatment (Figure 1C), which showed that triptolide damaged the interstitial tissue of rat testes. The protein levels of 3β-HSD and CYP11A1 (testosterone synthesis-associated enzymes) were significantly decreased after triptolide treatment (Figure 1C). However, the expression of nestin, an important marker of Leydig stem cells whose expression is increased after injury (Guo et al., 2013), was increased after triptolide treatment (Figure 1C). All these results demonstrate that triptolide damaged the interstitial tissue of rat testes, which might not be detectable by measuring testicular diameter or volumes.

### Triptolide Induces Rat Leydig Cell Apoptosis and Inhibits Leydig Cell Viability *In Vitro* and *In Vivo*

**FIGURE 2:**
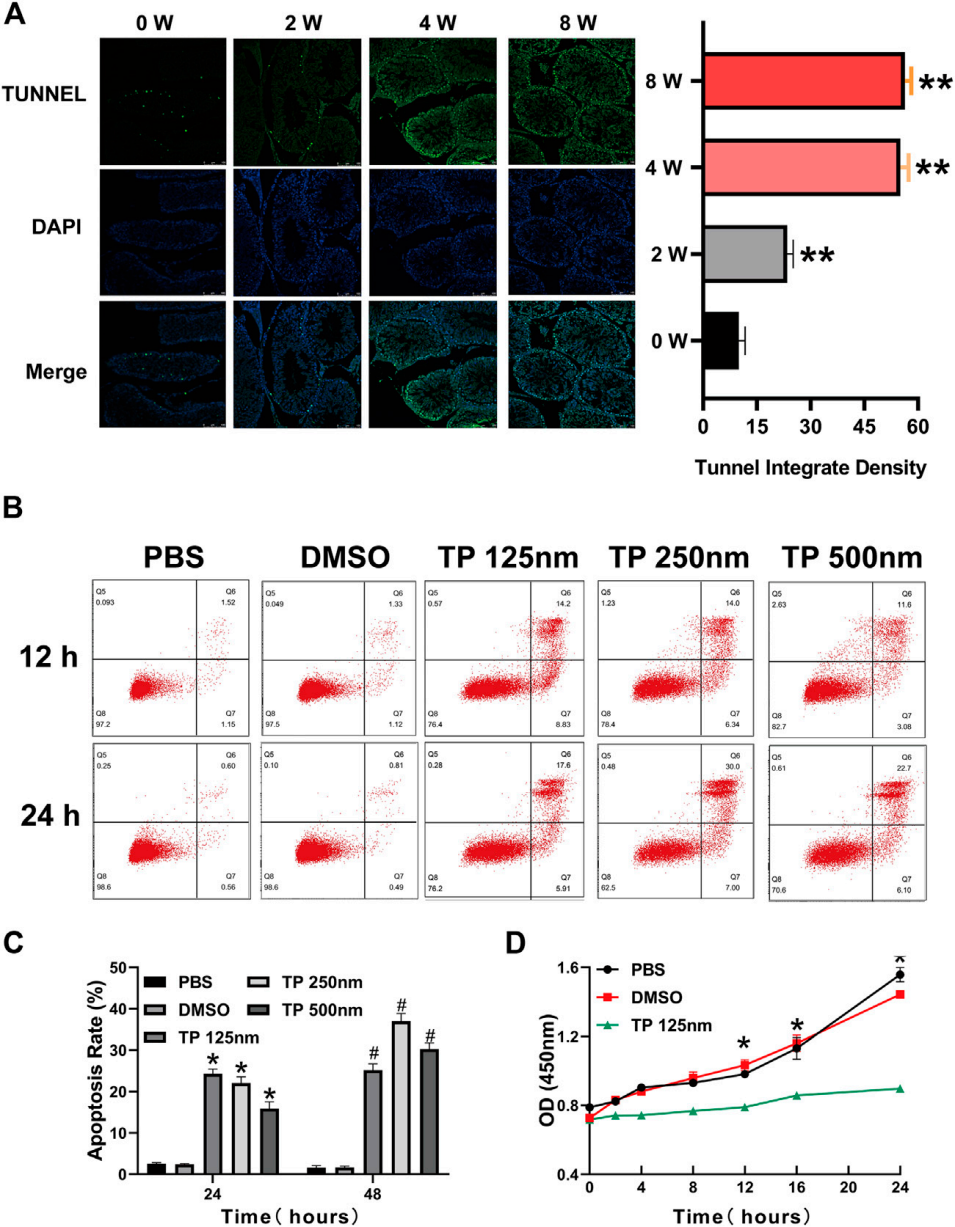
Triptolide induces Leydig cell apoptosis *in vitro* and *in vivo*. **(A)** Representative TUNEL staining images of rat testes. **(B)** Annexin V-FITC/PI apoptosis assays of TM3 cells. **(C)** Quantitative analysis of the results shown in **(B)**. **(D)** CCK-8 assay of TM3 cells. Data represent the mean ± SD from three independent experiments. Group comparisons were performed by one-way analysis of variance followed by Tukey’s post hoc test. **<0.01 vs. the DMSO group at 24 h, *p < 0.05 vs. the DMSO group at 24 h ^#^ *p* < 0.05 vs. the DMSO group at 48 h.

After we found that triptolide damaged the interstitial tissue of rat testes, we next investigated the cause of this damage. TUNEL staining of testis tissue showed a time-dependent increase in apoptotic cells in the interstitial tissue of the testis after triptolide treatment. One of the important cell types in the interstitial tissue of the testis is the Leydig cell, so we chose the Leydig cell line TM3 to determine the effect of triptolide *in vitro*. The results showed that triptolide induced apoptosis of TM3 cells (Figure 2B). Moreover, the CCK-8 assay showed that triptolide significantly decreased the viability of TM3 cells (Figure 2D).

3. Triptolide damages mitochondrial function and activates the mitochondrial apoptosis pathway *in vitro* and *in vivo*.

**FIGURE 3:**
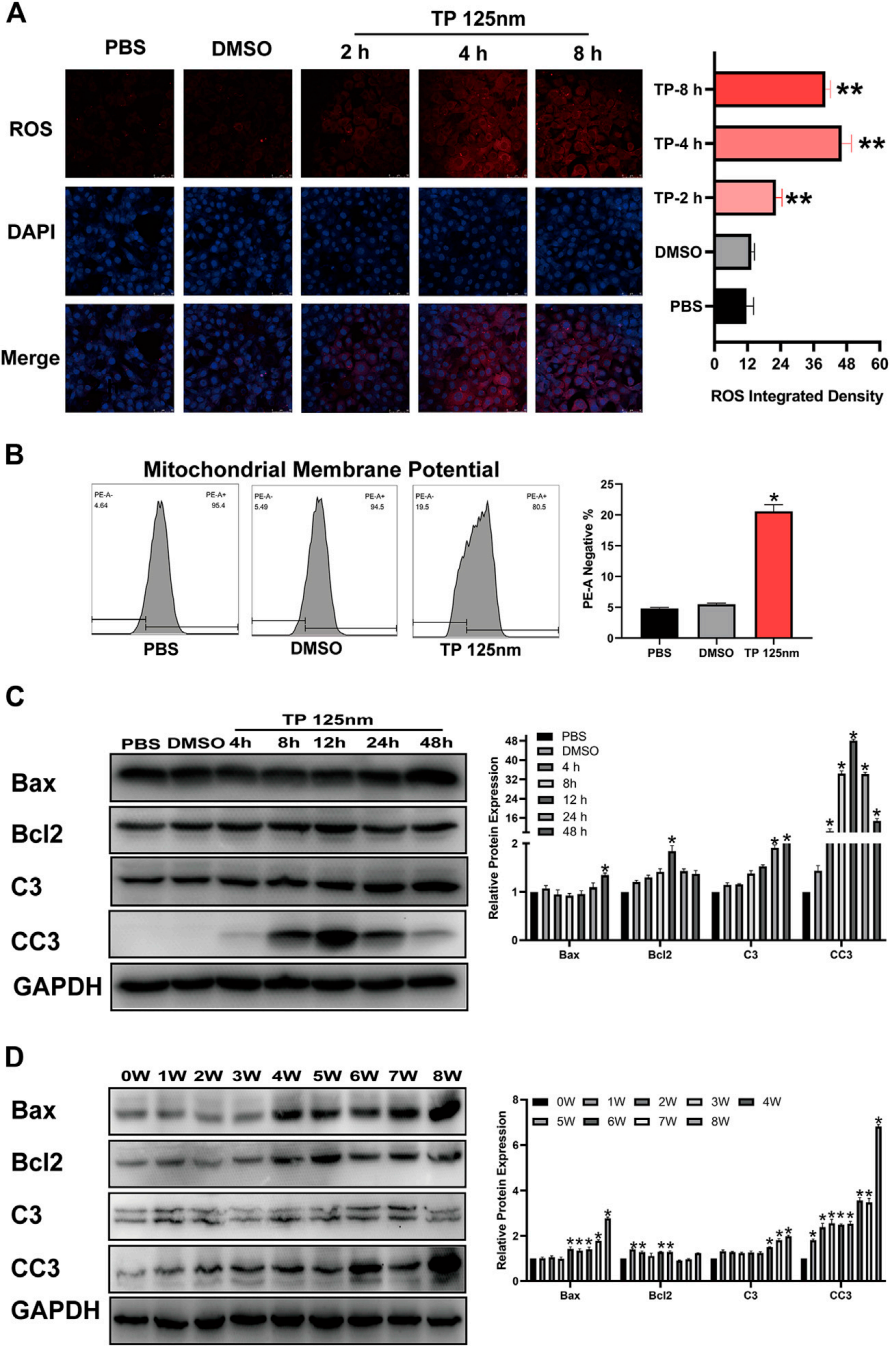
Triptolide induces mitochondrial dysfunction and activates the mitochondrial apoptosis pathway in TM3 cells. **(A)** ROS staining assay of TM3 cells. **(B)** Methyl tetramethylrhodamine assay of TM3 cells. **(C,D)** Western blot analysis of Bax, Bcl2, C3, and CC3 protein levels. GAPDH was used as a loading control. Data represent the mean ± SD from three independent experiments. Group comparisons were performed by one-way analysis of variance followed by Tukey’s post hoc test. *p < 0.05 vs. the DMSO or 0-week group.

Normal mitochondrial function is very important for Leydig cell survival, so we determined whether triptolide induces cell apoptosis by inducing mitochondrial dysfunction. The results showed that triptolide increased ROS levels (Figure 3A) and reduced mitochondrial membrane potential after triptolide treatment in TM3 cells (Figure 3B). Severe mitochondrial injury activates the mitochondrial apoptosis pathway and induces cell apoptosis, so we determined whether the mitochondrial apoptosis pathway is activated after triptolide treatment. The results showed that the expression levels of key factors in the mitochondrial apoptosis pathway, including Bax and CC3, were significantly increased *in vitro* and *in vivo* (Figures 3C,D), which indicates that mitochondrial apoptosis pathway activation may be the key step in triptolide-induced cell apoptosis.

### Triptolide Disrupts the Dynamic Mitochondrial Balance in Leydig Cells and Rat Testis

**FIGURE 4:**
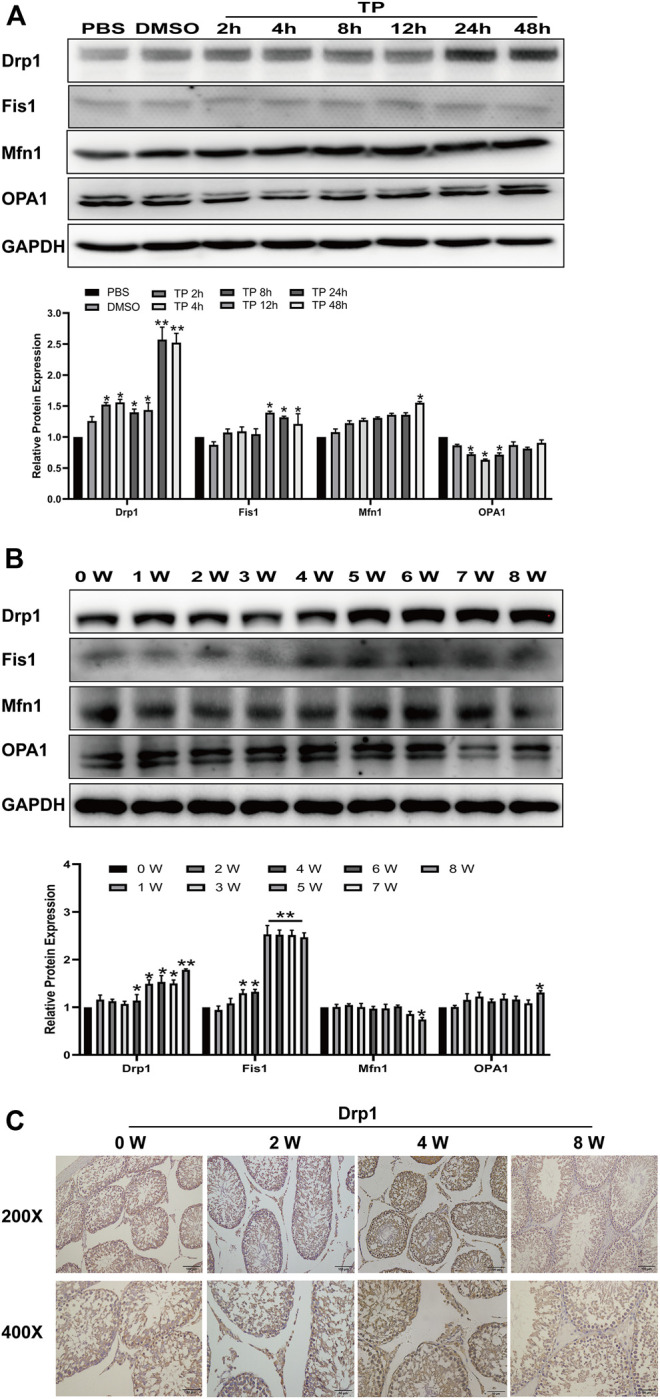
Triptolide increases Drp1 expression and disrupts mitochondrial dynamics *in vitro* and *in vivo*. **(A,B)** Western blot analysis of Drp1, Mfn1, OPA1, and Fis1 protein levels. GAPDH was used as a loading control. **(C)** Representative images of Drp1 immunostaining in rat testes. Data represent the mean ± SD from three independent experiments. Group comparisons were performed by one-way analysis of variance followed by Tukey’s post hoc test. **p < 0.01 vs. the DMSO or 0-week group. *p < 0.05 vs. the DMSO or 0-week group.

Next, we studied how triptolide causes mitochondrial dysfunction. Mitochondria are in a highly balanced state of fission and fusion, and disruption of this balance leads to mitochondrial dysfunction. Here, we found that triptolide significantly increased the expression of dynamin-related protein 1 (Drp1), a key factor in promoting mitochondrial fission, *in vitro* and *in vivo*, ultimately disrupting the dynamic balance of mitochondrial fission and fusion (Figures 4A,B). Immunohistochemical analysis of rat testis slices showed that the expression of Drp1 was increased in the interstitial tissue of rat testes (Figure 4C).

5. Mdivi-1 partially reverses the disruption of mitochondrial dynamic balance, mitochondrial dysfunction, and activation of the mitochondrial apoptosis pathway induced by triptolide.

**FIGURE 5:**
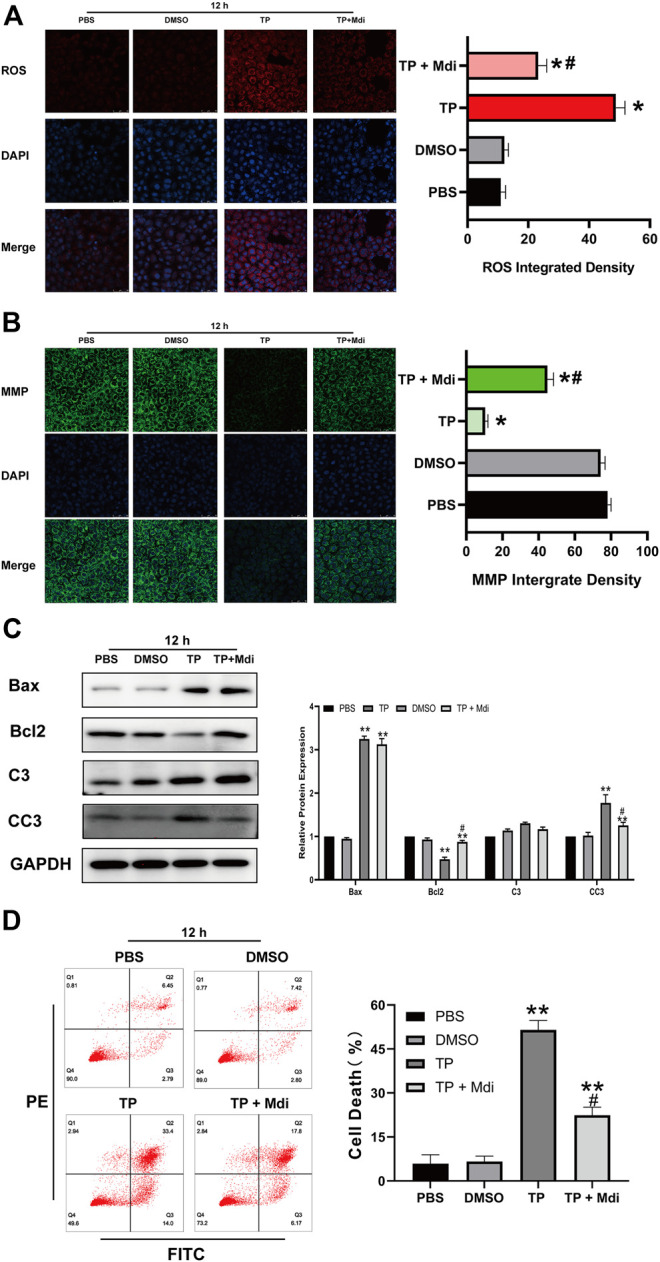
Mdivi-1 partially reverses triptolide-induced mitochondrial dynamic disruption, mitochondrial dysfunction and cell apoptosis *in vitro*. **(A)** ROS staining assay of TM3 cells. **(B)** Methyl tetramethylrhodamine assay of TM3 cells. **(C)** Western blot analysis of Bax, Bcl2, C3, and CC3 protein levels. GAPDH was used as a loading control. **(D)** Annexin V-FITC/PI apoptosis assays of TM3 cells. Data represent the mean ± SD from three independent experiments. Group comparisons were performed by one-way analysis of variance followed by Tukey’s post hoc test. **p < 0.01 vs. the DMSO or 0-week group. *p < 0.05 vs. the DMSO or 0-week group.

Triptolide causes mitochondrial dysfunction by disrupting the dynamic balance of mitochondrial fission and fusion, which activates the mitochondrial apoptosis pathway and finally induces cell apoptosis. We hypothesized that reversing the excessive mitochondrial fission induced by triptolide could preserve the normal mitochondrial function of cells and ultimately reduce cell apoptosis. We used Mdivi-1, a specific Drp1 inhibitor, to inhibit mitochondrial fission induced by triptolide. The results showed that Mdivi-1 reduced the expression of Drp1 (Figure 5A) and restored the dynamic balance of mitochondrial fission and fusion. Moreover, Mdivi-1 decreased the ROS level (Figure 5B), increased the mitochondrial membrane potential (Figure 5C), and inhibited mitochondrial apoptosis pathway activation after triptolide treatment (Figure 5D).

## Discussion

In the present study, we found that triptolide reduced testicular weight and serum testosterone in rats and induced Leydig cell apoptosis, mitochondrial dysfunction and interstitial tissue damage by disrupting the dynamic balance of mitochondrial fission and fusion. This effect was partially reversed by the dynamin-related protein-1 inhibitor Mdivi-1.

Triptolide is widely used in the clinic for its biological effects, which include anti-inflammatory, anti-rheumatoid, and anti-tumor effects (Liu, 2011). However, the reproductive toxicity of triptolide substantially limits its application in male patients with fertility needs. Triptolide was shown to cause infertility and abnormal spermatogenesis in male rats (Huynh et al., 2000; Huang et al., 2015). This effect was thought to be caused by triptolide directly damaging spermatogonia, but whether other cell types, including Leydig cells, participate in this process is not clear. Previous studies have shown that Leydig cells play an important role in testosterone production and spermatogenesis (Zirkin and Papadopoulos, 2018), and triptolide was shown to inhibit superoxide dismutase expression, which increased intracellular ROS in rat Leydig cells, finally inducing oxidative stress damage and cell death (Hu et al., 2015). In addition, triptolide increased miR-26a expression in rat Leydig cells, which decreased GSk 3β mRNA levels and inhibited testosterone production (Liang et al., 2019). Another study also reported that triptolide induced Leydig cell apoptosis, and this effect might be highly associated with miR-200 (Miao et al., 2020). These results suggested that miRNAs might play an important role in triptolide-induced Leydig cell apoptosis. In our study, we found that triptolide reduced testicular weight and serum testosterone levels but did not influence testicular volume. HE staining showed interstitial vacuolation and a significantly decreased cell number both inside and outside of the seminiferous tubule. Furthermore, we found that triptolide-induced Leydig cell death *in vivo* and *in vitro*, which might contribute to the decrease in serum testosterone. More interestingly, we observed that the protein levels of nestin, which is a marker of stem Leydig cells, increased after triptolide treatment. A previous study reported that stem Leydig cells could proliferate and differentiate into mature Leydig cells after Leydig cell loss (Guo et al., 2013; Chen et al., 2017). However, it is unclear whether triptolide promotes stem Leydig cell proliferation through other mechanisms; this possibility needs further exploration.

Mitochondria are very important for Leydig cell survival and testosterone production (Kong et al., 2017a). Previous studies have revealed that mitochondrial dysfunction of Leydig cells significantly reduced testosterone production and serum testosterone (Li et al., 2017a; Kong et al., 2017b; Goncalves et al., 2018). Here, we found that triptolide activated the mitochondrial apoptosis pathway, increased cell ROS, and depolarized mitochondrial membrane potential. According to these data, we conclude that triptolide damaged Leydig cell mitochondria and inhibited testosterone production, which might play a significant role in triptolide-induced sperm abnormalities.

The balanced state of mitochondrial dynamics, including mitochondrial fission and fusion, is crucial for normal mitochondrial function (Whitley et al., 2019). Research has shown that triptolide induces excessive activation of mitophagy, which leads to mitochondrial dysfunction and cell death (Hasnat et al., 2019). In addition, ageing-related mitochondrial dynamic disruption was associated with decreased serum testosterone (Sokanovic et al., 2020). Here, we found that triptolide significantly increased the expression of mitochondrial fission proteins, including dynamin-related protein 1 (drp1) and FIS1. Moreover, the expression of mitochondrial fusion proteins, including mitofusion 1 and optic atrophy type 1 (OPA1), was decreased after triptolide treatment. The changes in these proteins indicated that triptolide promoted mitochondrial fission and disrupted the balance of mitochondrial dynamics (Meyer et al., 2017; Tilokani et al., 2018). Because drp1 is the key factor in mitochondrial fission (Chandra et al., 2017; Ding et al., 2018), we explored whether inhibiting drp1 could alleviate triptolide-induced cell damage. Mitochondrial division inhibitor 1 (Mdivi-1) is a highly efficient Drp1 inhibitor that can mitigate excessive Drp1-dependent mitochondrial fission and subsequent cell apoptosis in different diseases (Fan et al., 2017; Ruiz et al., 2018; Wu et al., 2018). In our study, we found that Mdivi-1 reversed triptolide-induced mitochondrial dynamic instability to mitigate mitochondrial dysfunction, ultimately inhibiting cell apoptosis. These results suggest that triptolide disrupts the balance of mitochondrial dynamics in Leydig cells in a Drp1-dependent manner. Restoring the balance of mitochondrial dynamics may be an effective method to mitigate the reproductive toxicity of triptolide, and Drp1 is an ideal therapeutic target for this purpose. Highly efficient and specific inhibitors of Drp1 are needed.

## Data Availability

The original contributions presented in the study are included in the article/Supplementary Material, further inquiries can be directed to the corresponding authors.
