# Supplementary figures and images for: Triptolide Induces Leydig Cell Apoptosis by Disrupting Mitochondrial Dynamics in Rats
Source: Front Pharmacol. 2021 Mar 9;12:616803. doi: 10.3389/fphar.2021.616803 (PMC7985071; doi:10.3389/fphar.2021.616803)

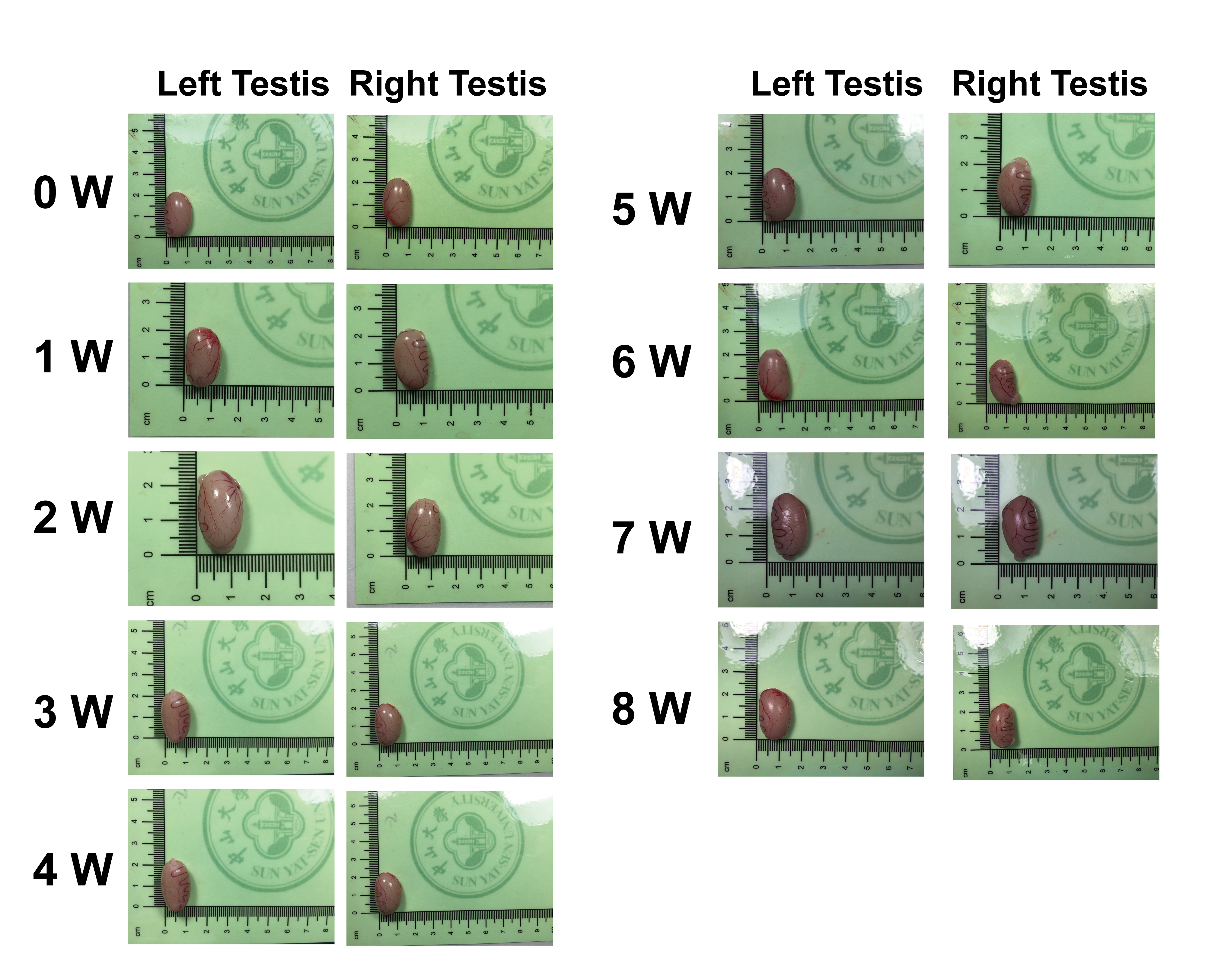

Supplement: Supplementary file 2 [file image2.tif]
